# *Caenorhabditis* nematodes colonize ephemeral resource patches in neotropical forests

**DOI:** 10.1002/ece3.9124

**Published:** 2022-07-24

**Authors:** Solomon A. Sloat, Luke M. Noble, Annalise B. Paaby, Max Bernstein, Audrey Chang, Taniya Kaur, John Yuen, Sophia C. Tintori, Jacqueline L. Jackson, Arielle Martel, Jose A. Salome Correa, Lewis Stevens, Karin Kiontke, Mark Blaxter, Matthew V. Rockman

**Affiliations:** ^1^ Department of Biology and Center for Genomics and Systems Biology New York University New York New York USA; ^2^ School of Biological Sciences Georgia Institute of Technology Atlanta Georgia USA; ^3^ Department of Molecular and Cell Biology University of California Berkeley California USA; ^4^ Renaissance School of Medicine Stony Brook University Stony Brook New York USA; ^5^ Tree of Life, Wellcome Sanger Institute Hinxton UK

**Keywords:** *Caenorhabditis*, dispersal, nematode, population biology, species description

## Abstract

Factors shaping the distribution and abundance of species include life‐history traits, population structure, and stochastic colonization–extinction dynamics. Field studies of model species groups help reveal the roles of these factors. Species of *Caenorhabditis* nematodes are highly divergent at the sequence level but exhibit highly conserved morphology, and many of these species live in sympatry on microbe‐rich patches of rotten material. Here, we use field experiments and large‐scale opportunistic collections to investigate species composition, abundance, and colonization efficiency of *Caenorhabditis* species in two of the world's best‐studied lowland tropical field sites: Barro Colorado Island in Panamá and La Selva in Sarapiquí, Costa Rica. We observed seven species of *Caenorhabditis*, four of them known only from these collections. We formally describe two species and place them within the *Caenorhabditis* phylogeny. While these localities contain species from many parts of the phylogeny, both localities were dominated by globally distributed androdiecious species. We found that *Caenorhabditis* individuals were able to colonize baits accessible only through phoresy and preferentially colonized baits that were in direct contact with the ground. We estimate the number of colonization events per patch to be low.

## Graphical abstract

*Caenorhabditis* nematodes are real animals that live in nature. Field collections and experiments in tropical forests reveal new information about their diversity, ecology, behavior, phylogenetics, population biology, and biogeography.

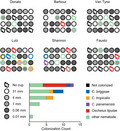

## 1 INTRODUCTION

*Caenorhabditis* (Osche, 1952) is diverse. High sequence divergence separates even closely related sister species (Dey et al., 2012; Ren et al., 2018). Species often live in sympatry, yet highly conserved morphology makes it difficult and in some cases impossible to distinguish them without the use of molecular tools or mating tests (Sudhaus & Kiontke, 2007). The morphological uniformity among species raises questions about their long‐term phenotypic stasis, species coexistence, and the niches they occupy. Previous studies of wild populations of *Caenorhabditis* find that they live on microbe‐rich patches of decaying fruit and vegetable matter (Crombie et al., 2019; Félix et al., 2013; Félix & Duveau, 2012; Ferrari et al., 2017; Frézal & Félix, 2015; Schulenburg & Félix, 2017), the stages on which niche partitioning and interspecific competition play out. Stochastic colonization and extinction rates on these ephemeral resources are key parameters in understanding the local coexistence of species (Dubart et al., 2019).

Some *Caenorhabditis* species occupy a substrate niche as specialists (Dayi et al., 2021; Kanzaki et al., 2018; Li et al., 2014). The majority, however, have no obvious substrate preference. Studies of *Caenorhabditis* microbiomes in both the laboratory (Berg et al., 2016) and the field (Dirksen et al., 2016; Zhang et al., 2017) suggest that animals regulate the composition of their gut flora on substrates with differing microbial composition. From these data, one could hypothesize that species with overlapping ranges specialize by occupying niches defined by what they eat. However, it is unclear which microbes are the primary food source of worms in the wild (Schulenburg & Félix, 2017). Beyond food, other factors including predators and pathogens along with nonbiological sources of variation like humidity and temperature may play a role in determining where *Caenorhabditis* species colonize and proliferate (Crombie et al., 2019; Félix & Duveau, 2012). Still missing is substantial evidence that these *Caenorhabditis* species preferentially colonize substrates like fruits versus flowers. However, one field study found the degree to which a patch is rotting may influence the incidence of species found on those patches, suggesting that priority effects and ecological succession may also be involved in species coexistence (Ferrari et al., 2017).

Equally critical to understanding *Caenorhabditis* species adaptations to ephemeral resource patches are determining modes of dispersal. Two models described by Slatkin (1977) represent the extremes of a theoretical spectrum. In the *propagule pool* model, all colonists are derived from a single patch, whereas in the *migrant pool* model, colonizers come from every patch in the metapopulation. In *Caenorhabditis*, these models encompass potential modes of dispersal, either by a phoretic host (Kiontke, 1997; Sudhaus et al., 2011; Woodruff & Phillips, 2018; Yoshiga et al., 2013) or by a semimobile “seed bank” of dauer larvae crawling towards or waiting for a fresh patch (Cutter, 2015). These contrasting modes of dispersal may have profound effects on the level of inbreeding and genetic diversity (Li et al., 2014). In addition, propagule size may contribute to the evolution of a female‐biased sex ratio and the evolution of self‐fertile hermaphroditism as a means of generating a population growth advantage and reproductive assurance, respectively (Cutter et al., 2019; Hamilton, 1967; Lo et al., 2021; Theologidis et al., 2014).

To better understand *Caenorhabditis* diversity and the factors that influence it, we performed field surveys and experiments in two of the most intensively studied lowland tropical forests on Earth: Barro Colorado Island (BCI), Panamá, and La Selva, Costa Rica. Barro Colorado Island lies in the center of the Panama Canal in the man‐made Lake Gatún. Shortly after its formation, the island was designated a protected nature reserve and has been hosting field research for the last 100 years (Leigh, 1999). Likewise, La Selva Biological Field Station in Sarapiquí, Costa Rica, has been a protected research forest for nearly 70 years (McDade et al., 1994). We focused our collection efforts on these two localities as they are relatively undisturbed by human activity, and their histories of intensive research provide a rich source of information about the local ecology. One nematode metagenetic study previously found *Caenorhabditis* DNA in a soil and leaf litter sample at BCI, but the species were not identified (Porazinka et al., 2010). In contrast to the majority of previous work on *Caenorhabditis* in the tropics, which involved transporting substrates out of the country and isolating animals from nematode growth medium plates days or weeks later, we isolated and cultured all animals immediately in the field, potentially reducing sampling biases that favor species that survive transport and grow well on nematode growth medium. One other study used a combination of these approaches (Félix et al., 2013).

In total, we collected seven species of *Caenorhabditis*, four of them known only from these collections. We formally describe two new species, *C. krikudae* sp. nov. and *C. agridulce* sp. nov., and we place them within the *Caenorhabditis* phylogeny. Each locality was dominated by globally distributed self‐fertile species. We assayed several ecological features related to patch accessibility, patch specificity, and co‐occurrence of species. Using baits that vary in their accessibility, we demonstrate that *Caenorhabditis* are able to colonize baits that are only accessible by phoresy. Further, the colonization rate varied significantly with accessibility where baits making direct contact with the ground were preferentially colonized. We found that individual species tended to occur in habitat patches close to other patches of conspecifics, and we use the frequency of uncolonized patches to estimate the number of colonization events per patch. Taken together, our data support a model that many *Caenorhabditis* species are habitat generalists whose population biology is strongly influenced by metapopulation dynamics.

## 2 MATERIALS AND METHODS

### 2.1 Collections

We collected nematodes on BCI in May 2012 (wet season), March 2015 (dry season), and August 2018 (wet season). Schemes for sampling varied within and among sampling sessions and included opportunistic sampling and the use of baits as described in the results. In all cases, worms were isolated from substrates and transferred to Nematode Growth Medium (NGM) plates at the BCI field station and identified as *Caenorhabditis* by morphology under a stereomicroscope. In 2012 and 2015, material from the forest (e.g., rotting fruits and flowers) was placed directly onto NGM plates, and *Caenorhabditis* worms were picked to new plates to establish cultures (Barrière & Félix, 2005). These individual patches of organic material are defined as samples in our dataset and were evaluated for the presence of nematodes. For the majority of samples collected in 2018, worms were isolated by the Baermann funnel technique (Baermann, 1917; Tintori et al., 2022) and subsequently cultured on NGM plates. These cultures were transported to New York for species determination as described below.

We collected nematodes at La Selva, Costa Rica, in July 2019, by Baermann funnel. We used two methods to identify *Caenorhabditis* to species. Individual *Caenorhabditis* worms were chopped with razor blades, transferred to Whatman paper as described by Marek et al., 2014, and transported to New York. There, the stored nematode DNA was used to identify the specimen to species by ITS2 sequencing. Separately, we established isofemale cultures on NGM plates. These plates were stored at La Selva for six months prior to their transport to New York, where surviving cultures were revived and species identified as described below. Complete collection data are reported in Supporting Information File 1.

### 2.2 Species identification

Species were identified by sequencing a fragment of rDNA to derive a prediction. Subsequently, experimental crosses were performed with isolates of known species identity to establish a biological species assignment (Félix et al., 2014; Ferrari et al., 2017; Kiontke et al., 2011; Stevens et al., 2019).

For sequence‐based predictions, individuals were picked individually into 30–50 μl of Worm Lysis Buffer (50 mM KCL, 2.5 mM MgCl2, 10 mM Tris pH 8.3, 0.45% IGEPAL, 0.45% Tween‐20, 0.01% Gelatin, 2 mg/ml proteinase K) and freeze‐cracked for 10 min at −80°C followed by a 90‐minute digestion at 65°C and a 95°C heat inactivation for 15 min. These lysates or nematode DNA stored on Whatman paper were used as templates for PCR. Amplifications included PCRs using G18S4a (5′‐GCTCAAGTAAAAGATTAAGCCATGC) and DF18S‐B (5′‐YGATCCABCBGCAGGTTC) to amplify a 1 kb region of the 18S ribosomal DNA (Kiontke et al., 2004), PCRs using 5.8S‐1 (5′‐CTGCGTTACTTACCACGAATTGCARAC) and KK28S‐4 (5′‐GCGGTATTTGCTACTACCAYYAMGATCTGC) to amplify a 2 kb region around the ITS2 region (Kiontke et al., 2011), and PCRs using RHAB1350F (5′‐TACAATGGAAGGCAGCAGGC) and RHAB1868R (5′‐CCTCTGACTTTCGTTCTTGATTAA) to amplify a fragment of about 500 bp of 18S (Haber et al., 2005). After Sanger sequencing the PCR amplicons, we performed BLAST searches (Camacho et al., 2009) against the NCBI GenBank database or compared the sequences to our database of *Caenorhabditis* rDNA sequences to find the closest match.

Mating tests were performed with worms of known species identity. Cross plates were monitored for the presence of viable progeny. For isolates of androdioecious species, hermaphrodites were crossed to males from strains of *C. briggsae* and *C. tropicalis*. In some cases, we used males of wild‐type strains AF16 and JU1373, respectively, and monitored plates for male progeny. In other cases, we used males of strains QG2801, an AF16 derivative carrying GFP‐expressing transgene *syIs803* (Inoue et al., 2007), and QG3501, a derivative of *C. tropicalis* NIC58 carrying mCherry‐expressing transgene *qgIs5* (Noble et al., 2021), and we monitored for wild‐type green‐ or red‐fluorescent offspring.

### 2.3 Sequencing and assembling the transcriptome of *Caenorhabditis krikudae* n. sp.

We generated the *C. krikudae* n. sp. inbred line QG3077 by 28 generations of full‐sibling mating from isofemale line QG3050. We generated RNA‐seq mRNA transcriptome data using a pool of five mixed‐stage populations of QG3077, with each population being subjected to a different condition. All worms were grown at 25°C on 10 cm NGMA plates (for 1 L: 3 g NaCl, 5 g bacto‐peptone, 10 g agar, 7 g agarose, 1 ml cholesterol 5 mg/ml in ethanol, 1 ml CaCl_2_ 1 M, 1 ml MgSO_4_ 1 M, 25 ml KPO_4_ 1 M). One population was fed with CemBio strains (Dirksen et al., 2020), and the other four were fed with *E. coli* OP50. The conditions for OP50 populations consisted of (1) mixed‐stage, (2) starved, (3) heat‐stressed, and (4) cold‐stressed. Temperature stress consisted of exposing the worms to either 35°C or 4°C for 2 h followed by a 2‐hour recovery prior to RNA extraction. Total RNA was isolated using TriZol following the protocol described in Green and Sambrook (2020). The mRNA library was constructed using the Illumina Stranded mRNA Prep Ligation protocol. The library was sequenced using a NextSeq 500 MidOutput 2X150 for 300 cycles. Paired‐end sequences were trimmed with Trim Galore (https://github.com/FelixKrueger/TrimGalore). Trimmed sequences were assembled into a transcriptome using Trinity (Grabherr et al., 2013) also running default parameters for paired‐end reads. We then generated the longest predicted ORFs using TransDecoder (https://github.com/TransDecoder/TransDecoder) for use in phylogenetic analyses.

### 2.4 Sequencing and assembling the genome and transcriptome of *Caenorhabditis agridulce* n. sp.

Isofemale strain QG555 was grown on 9 cm NGMA plates. We harvested nematodes just after starvation and washed using M9 several times to remove *E. coli*. For genomic DNA extraction, the nematode pellets were suspended in 600 μl of Cell Lysis Solution (Qiagen) with 5 μl of proteinase K (20 μg/μl) and incubated overnight at 56°C with shaking. The following day, the lysate was incubated for one hour at 37°C with 10 μl of RNAse A (20 μg/μl) and the proteins were precipitated with 200 μl of protein precipitation solution (Qiagen). After centrifugation, we collected the supernatant in a clean tube and precipitated the genomic DNA using 600 μl of isopropanol. The DNA pellets were washed in 70% ethanol and dried for one hour before being resuspended in 50 μl of DNAse‐free water. For RNA extraction, we resuspended 100 μl of nematode pellet in 500 μl of Trizol (5 volumes of Trizol per volume of pelleted nematodes). The Trizol suspension was frozen in liquid nitrogen and then transferred to a 37°C water bath to be thawed completely. This freezing/thawing process was repeated four to five times and the suspension was vortexed for 30 s and let rest for 30 s (five cycles). A total of 100 μl chloroform was added and the tubes were shaken vigorously by hand for 15 sec and incubated for 2–3 min at room temperature. After centrifugation (15 min at 13,000 rpm and 4°C), the aqueous (upper) phase containing the RNA was transferred to a new tube and precipitated with 250 μl of isopropanol. The pellets were washed in 70% ethanol and dried for 15–20 min before being resuspended with 50–100 μl of RNAse‐free water. An aliquot of each DNA and RNA preparation was run on agarose gel to check their quality and quantitated with Qubit (Thermo Scientific). Two short‐insert (insert sizes of 300 and 600 bp, respectively) genomic libraries and a single short‐insert (150 bp) RNA library were prepared using Illumina Nextera reagents and sequenced (125 bases, paired‐end) on an Illumina HiSeq 4000 at Edinburgh Genomics (Edinburgh, UK). All raw data have been deposited in the relevant International Nucleotide Sequence Database Collaboration (INSDC) databases.

We performed quality control of our genomic and transcriptomic read sets using FastQC (v0.11.9; Andrews, 2010) and used fastp (0.20.1; Chen et al., 2018 *; ‐‐length_required 50*) to remove low‐quality bases and Illumina adapter sequence. We generated a preliminary genome assembly using SPAdes (v3.14.1; Bankevich et al., 2012; *‐‐only‐assembler ‐‐isolate ‐k 21,33,55,77*) and identified the likely taxonomic origin of each contig by searching against the NCBI nucleotide (nt) database using BLASTN (2.10.1+; Camacho et al., 2009; *−task megablast ‐max_target_seqs 1 ‐max_hsps 1 ‐evalue 1e‐25*) or by searching against UniProt Reference Proteomes database using Diamond BLAST (2.0.4; Buchfink et al., 2015; *‐‐max‐target‐seqs 1 ‐‐sensitive ‐‐evalue 1e‐25*). We also mapped the genomic reads to the genome assembly using bwa mem (0.7.17‐r1188; Li, 2013). We provided the assembly, the BAM file, and the BLAST and Diamond files to blobtools (1.1.1; Laetsch & Blaxter, 2017) to generate taxon‐annotated, GC‐coverage plots, which we used to identify contaminant contigs. Any read pair that mapped to the contaminant contigs was discarded. Using this filtered read set, we generated a final assembly using SPAdes (*‐‐isolate ‐k 21,33,55,77,99*). We also generated a transcriptome assembly using Trinity (Trinity‐v2.8.5; Haas et al., 2013), which we then used to scaffold the genome assembly using SCUBAT2 (available at https://github.com/GDKO/SCUBAT2). We used numerical metrics and BUSCO (v4.1.4; Simão et al., 2015; *−l nematoda_odb10 ‐m genome*) to assess assembly quality and biological completeness, respectively. Prior to gene prediction, we generated a species‐specific repeat library using RepeatModeler (2.0.1; Smit & Hubley, 2010; *‐engine ncbi*), and combined this library with known Rhabditid repeats from RepBase (Jurka et al., 2005). This repeat library was then used to soft‐mask the genome using RepeatMasker (open‐4.0.9; Smit et al., 1996; *−xsmall*). We predicted genes in the genome by aligning trimmed transcriptomic data to the genome using STAR (2.7.3a; Dobin et al., 2013 *; ‐twopassMode Basic*) and providing the resulting BAM file to BRAKER2 for gene prediction (2.1.5; Brůna et al., 2021; *‐‐softmasking*). We used BUSCO (*−l nematoda_odb10 ‐m proteins*) to assess gene set completeness.

### 2.5 Phylogenetic analysis

We identified a set of orthologous proteins by running BUSCO (Seppey et al., 2019) using the nematode_odb10 dataset on each of the 36 nematode genomes found in Table S1. Multisequence fasta files for each ortholog were extracted using busco2fasta (https://github.com/lstevens17/busco2fasta) with the setting ‐p 0.8, meaning each ortholog was required to be in 80% or 28 of the 36 species. Orthologous sequences were then aligned with MAFFT (Katoh & Standley, 2013) and ML gene trees estimated using IQ‐TREE (Nguyen et al., 2015), both on default settings. Newick trees were concatenated into a single file and a species tree was estimated using ASTRAL‐III (C. Zhang et al., 2018), which uses a coalescent framework. We also generated a species tree using a supermatrix of all concatenated orthologs. To generate the supermatrix we used TrimAl (Capella‐Gutiérrez et al., 2009) to remove poorly aligned regions using the settings ‐gt 0.8 ‐st 0.001 ‐resoverlap 0.75 ‐seqoverlap 80. Sequences were subsequently concatenated using catfasta2phyml (https://github.com/nylander/catfasta2phyml). A tree was then inferred with IQ‐TREE using the LG substitution model (Le & Gascuel, 2008), modeling the rate variation among sites using a Discrete Gamma model (Yang, 1994) with 4 categories. Support was estimated using 1000 ultrafast bootstrap replicates (Hoang et al., 2018). We then estimated ASTRAL‐III tree branch lengths in units of replacements per site rather than coalescent units using IQ‐TREE with the same parameters as the supermatrix analysis while fixing the tree by the output of the ASTRAL‐III analysis using the ‐te setting. All newick trees were visualized using the ITOL web browser (Letunic & Bork, 2019).

## 3 RESULTS

### 3.1 The *Caenorhabditis* faunas of BCI and La Selva

**FIGURE 1:**
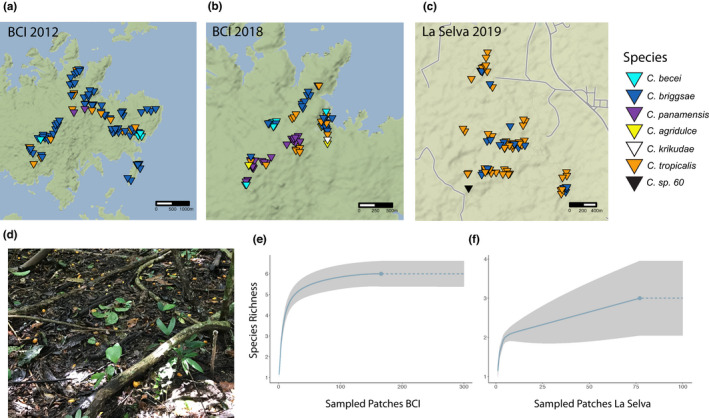
Collection sites for *Caenorhabditis* species used in this study. *Caenorhabditis* were collected at two localities: Barro Colorado Island, Panamá, and La Selva, Sarapiquí, Costa Rica. (a–c) Distribution of species collected from opportunistic sampling from each locality by year. Each marker represents a patch positive for that species. Patches may be plotted multiple times if species co‐occurred on the same patch. Patches are jittered to prevent overpotting. (d) A field of rotting *Spondias mombin* substrates (e,f). Rarefaction curve of the chao2 incidence‐based estimator for both localities. The solid line represents the predicted species richness the dotted line represents an extrapolation of species richness. The gray area is the 95% confidence interval.

We recovered *Caenorhabditis* nematodes from 225 samples collected on BCI (Figure 1; Supporting Information File 1). Additional samples did not contain *Caenorhabditis* or were damaged during processing and shipping. The *Caenorhabditis* isolates derive from opportunistic sampling of rotten fruits, flowers, mushrooms, and leaf litter in 2012 and 2018, from a systematic sampling of *Gustavia superba* flowers in 2012, and from several classes of experimental baits in 2015. By DNA barcode sequencing and laboratory mating tests, we assigned the *Caenorhabditis* isolates to six different species, three of which are currently known only from our collections on BCI. These are *C. becei* Stevens 2019, *C. panamensis* Stevens 2019, and *C*. *krikudae* n. sp, which we formally describe in the Appendix to this paper. The number of samples yielding each species is shown in Table 1. In total, the 225 samples yielded 260 species observations, as many samples contained multiple *Caenorhabditis* species.

**TABLE 1:** Species counts for samples collected on BCI and positive for *Caenorhabditis*. The 2018 survey is a subset collected by a single investigator.

Species	Total positive samples	2018 Survey	Range
*C. briggsae*	152	26	Cosmopolitan
*C. tropicalis*	43	15	Pantropical
*C. panamensis*	30	11	Endemic
*C. becei*	25	10	Endemic
*C. agridulce*	8	6	Neotropical
*C. krikudae*	2	1	Endemic

To estimate the frequency of *Caenorhabditis* across samples while minimizing variation due to differences in sampling technique, we consider a dataset of 177 samples collected and processed by a single investigator in August 2018. These samples included a range of rotten fruits, flowers, stems, fungi, and leaf litter. Overall, 94% of the samples yielded nematodes, and 32% (57/177) yielded *Caenorhabditis*. Some samples again contained multiple *Caenorhabditis* species, resulting in 69 species observations (Table 1).

To assess the completeness of our survey, we used rarefaction of the chao2 incidence‐based estimator (Chao et al., 2014; Hsieh et al., 2016), which generated an estimated species richness of 6 ± 0.34 (95% CI) (Figure 1). These data suggest that we have recovered the maximum number of species at BCI, conditional on our sampling strategy. The two most abundant species, *C. briggsae* and *C. tropicalis*, are androdioecious (males and self‐fertile hermaphrodites), and their geographic distributions are cosmopolitan and pantropical, respectively. The other species are gonochoristic (males and females). One of these species, *C. agridulce* n. sp., which we formally describe in the Appendix to this paper, has also been found in French Guiana (Ferrari et al., 2017), Mexico, and Southern California (Appendix 1).

We successfully recovered *Caenorhabditis* nematodes from 77 samples at La Selva, Costa Rica (Figure 1; Supporting Information File 1). These derive from an opportunistic sampling of rotten fruits, flowers, mushrooms, and litter in 2019. These samples yielded only 3 different species, one of which is known only from our collections at La Selva (*C*. sp. 60). La Selva differed from BCI in that *C. tropicalis* was most prevalent (present in 55 samples), followed by *C. briggsae* (32 samples). Gonochoristic *C*. sp. 60 was isolated from a single substrate, which contained thousands of individuals. The rarefaction of the chao2 incidence‐based estimator generates a species richness of 3 ± 0.48 (95% CI) (Figure 1). This suggests that the lower number of observed species at La Selva is not due to inadequate sampling given our sampling strategy. We measured substrate temperature for 22 samples that contained *Caenorhabditis*; these ranged from 24.1 to 28.4°C. There was no difference in temperature between patches containing different species, with a mean temperature of 26°C for each species (Supporting Information File 1).

**FIGURE 2:**
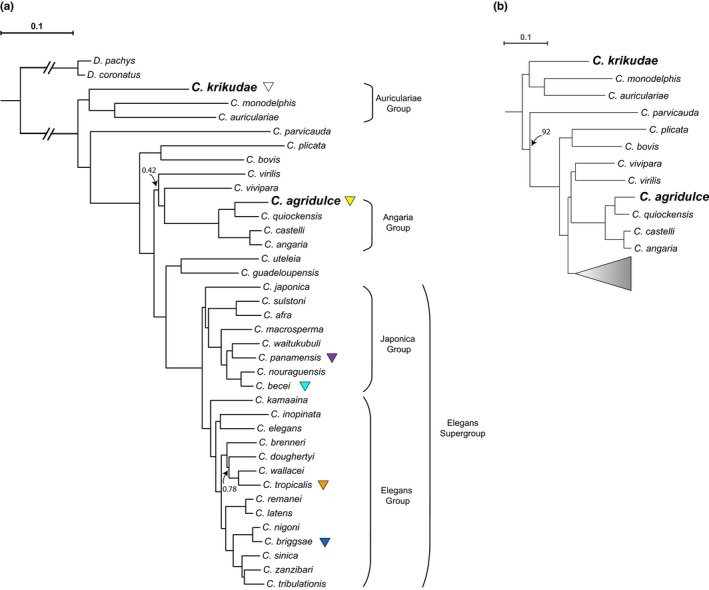
Phylogeny of 36 *Caenorhabditis* species, with *D. coronatus* and *D. pachys* forming an outgroup, based on 1931 single‐copy orthologs each shared between 80% of the species. (a) Phylogeny inferred using a coalescent approach that takes gene trees as input (substitution models for each gene tree selected automatically). Branch lengths in substitutions per site were estimated using the LG substitution model with gamma‐distributed rate variation among sites (LG + Γ) while fixing the phylogeny to the coalescent tree topology. Species incorporated into the phylogeny for the first time are bolded. Posterior probabilities are 1.0 unless noted. (b) Alternative topology using a supermatrix approach that uses concatenated alignments of all orthologs as input under an LG + Γ model. Bootstrap support is 100 unless noted.

To understand the phylogenetic positions of the undescribed species, we sequenced and assembled transcriptome for *C. krikudae* n. sp. and a genome for *C. agridulce* n. sp. Using these assemblies and the assemblies of 34 additional *Caenorhabditis* species, we identified 1931 single‐copy orthologs that were represented in at least 28 of the 36 species. We reconstructed the *Caenorhabditis* phylogeny using two approaches. First, we used a coalescent‐based approach with individual gene trees as input. Second, we used a maximum likelihood approach using a concatenated alignment of all orthologues as input. The resulting phylogenies (Figure 2) exhibit largely congruent topologies that are consistent with previous analyses (Stevens et al., 2019), differing only in the position of *C. virilis*. *C*. *agridulce* n. sp. is closely related to *C. quiockensis* within the *Angaria* group of spiral‐mating species (Sudhaus et al., 2011). *C. krikudae* n. sp. is most closely related to *C. monodelphis* and *C. auriculariae*,

which together form the sister group to all other *Caenorhabditis*. We name this clade the Auriculariae group, defined as species more closely related to *C. auriculariae* than to *C. elegans*. Based on data from ITS2 sequence only, *C*. sp. 60 is sister to *C. macrosperma* within the *Japonica* group (NCBI Accession: OL960095).

Overall, the species found at BCI and La Selva span the *Caenorhabditis* phylogeny. The two androdiecious species, *C. briggsae* and *C. tropicalis*, are the sole representatives of the *Elegans* group, while three species (*C. becei*, *C. panamensis*, *C*. sp. 60) are members of a neotropical‐endemic clade within the *Japonica* group.

### 3.2 Substrate specificities

To test whether the common *Caenorhabditis* species show substrate specificity, we analyzed the dataset of 177 samples processed by a single investigator in 2018 (Table 1, Supporting Information File 1). Each of the four most common species was collected from multiple types of fruit and flower. Classifications of the substrates, at high levels (fruit vs. other) or lower levels (fruit type), revealed no significant association between *Caenorhabditis* generally or any of the common species specifically and any substrate (logistic regression, *p* > .05; see Supporting Information File 1 for specific values). Acknowledging the very limited statistical power of these tests, we interpret this as evidence that the common species are substrate generalists, colonizing and proliferating in any available habitat patch.

### 3.3 The spatial patterning of patch occupancy

To understand the spatial patterning of *Caenorhabditis* among habitat patches, we performed a hierarchical spatial sampling of a single substrate type, rotten flowers of *Gustavia superba*, in May 2012. We selected four *G. superba* trees spread across the island. At each tree, we established three well‐separated 1 m^2^ quadrats. Within each quadrat, we sampled four rotten flowers, each at least 10 cm apart. From each flower that yielded *Caenorhabditis*, we established isofemale or isohermaphrodite lines from four or more randomly selected worms from each flower. At one tree, only two quadrats were sampled. In total this sampling scheme involved 44 samples of *G. superba* flowers.

**FIGURE 3:**
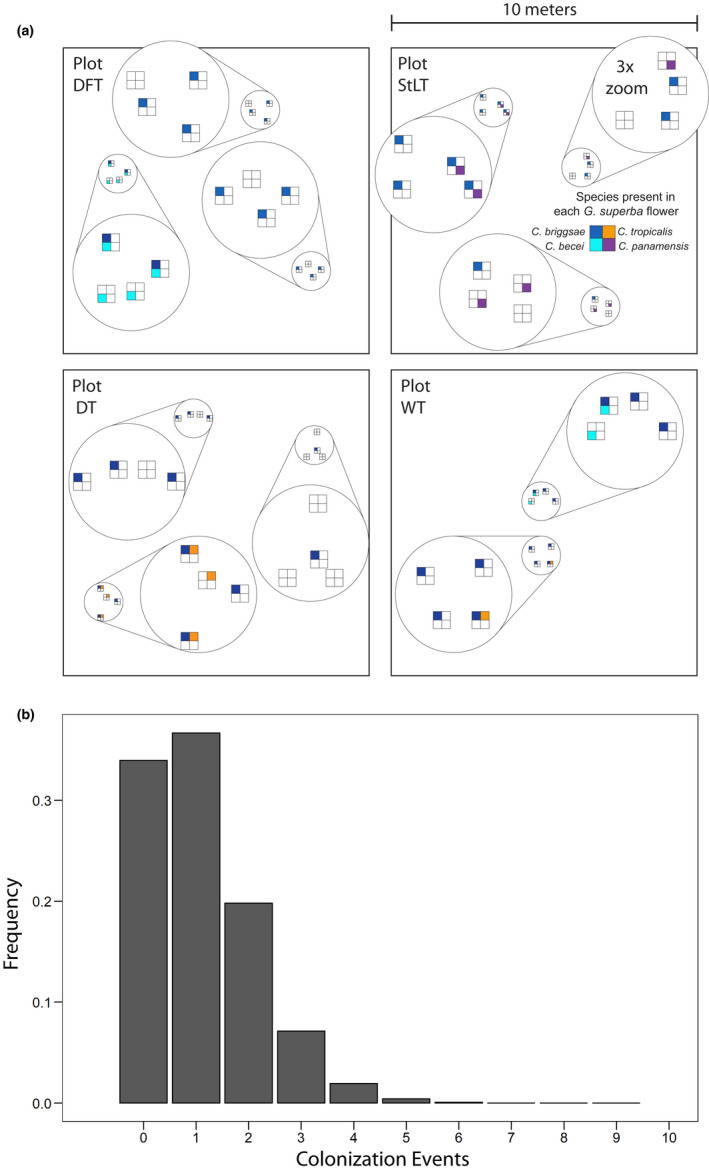
Species are patchily distributed among rotting *Gustavia superba* flowers. (a) 10 × 10 meter plots were systematically sampled at each of four focal trees. At each plot, four flowers were collected from two or three 1‐meter quadrats. Each box represents a flower; each color represents the species present on that flower (b). The distribution of *C. briggsae* colonization events per flower under a simple Poisson model (mean = 1.08).

Thirty‐six of 44 *G. superba* flowers (82%) contained *Caenorhabditis*. *C. briggsae* was present in every *Caenorhabditis*‐positive quadrat at every site, while the other species exhibited strongly patchy distributions over scales of meters (Figure 3). For example, *C. becei* was present in all four flowers in one quadrat at Plot DFT but absent from the flowers in the other two quadrats there. Similarly, *C. tropicalis* was present in three of four flowers in one quadrat at Plot DT but absent from the other two quadrats a few meters away. This patchiness is manifest at larger scales as well: *C. panamensis* was present in all three quadrats at Plot StLT but absent from the other three plots.

*C. briggsae* was present in 29 of the 44 *G. superba* flowers (66%). This allows a crude estimate of the number of flowers colonized by *C. briggsae* multiple times. If *C. briggsae* is present ubiquitously and patch colonization is a Poisson process, the absence of *C. briggsae* from 34% of flowers implies a Poisson‐distributed number of colonizations per patch with a mean of 1.08, with 29% of flowers colonized by *C. briggsae* more than once. Thus ~44% of the flowers that contained *C. briggsae* (0.29/0.66) are expected to have had multiple colonizations.

There is no evidence that the presence of one species affects the probability of observing a second species within a sample. For example, *C. briggsae* and *C. tropicalis* are present in 66% and 9% of the 44 samples; the expected co‐occurrence under independence is 2.6/44 and we observe co‐occurrence of 3/44 samples.

### 3.4 Colonization patterns among classes of bait

**FIGURE 4:**
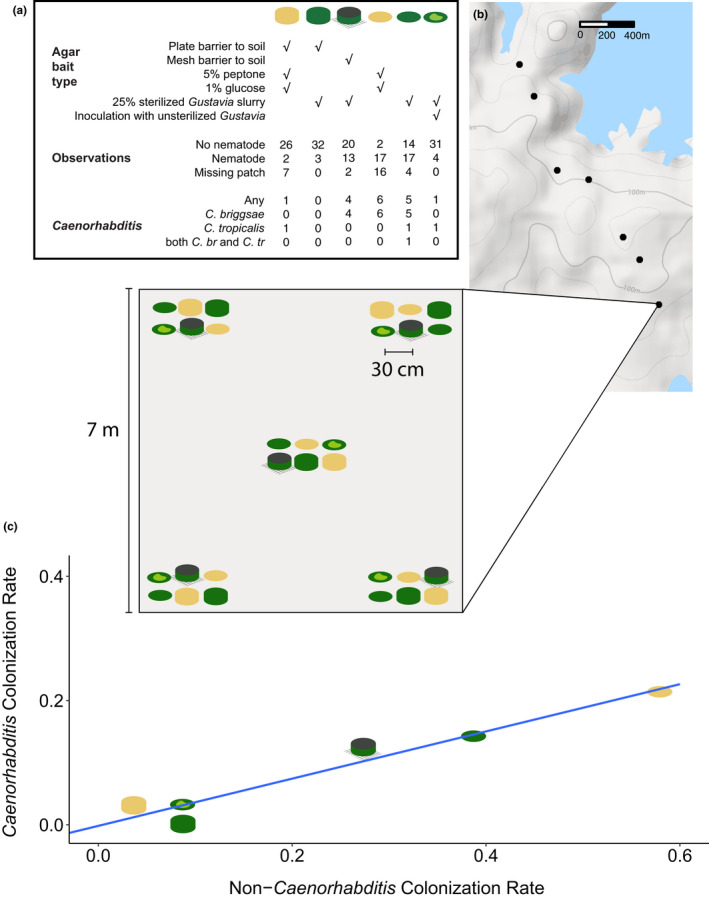
Colonization rates vary in response to bait composition and accessibility. (a) Table describing the six types of baits used in the experiment, the observations for each of the baits, and the counts of *Caenorhabditis*‐positive baits. (b) Baits were set up at each of seven sites across BCI. Each site consisted of 30 baits arranged in groups of six in the corners and center of each site. (c) The six types of agar bait showed different rates of colonization by nematodes. The blue line is linear regression of *Caenorhabditis* on non‐*Caenorhabditis* colonization rates across bait types.

To test how substrate type and accessibility affect rates of colonization by *Caenorhabditis*, we set up arrays consisting of several bait types. At each of the seven sites on BCI, we set up a 7‐by‐7‐meter field site with five arrays of baits (four in the corners, one in the center). Each bait array consisted of six agar baits, each bait of a different type (Figure 4), arranged 3 × 2 with 30 cm spacing between the 6‐cm diameter baits. Our experiment as a whole therefore included 210 baits in total.

Two of the bait types consisted of 1% agar supplemented with 5% peptone and 1% glucose. In one such bait type, the agar was placed on the forest floor in its plastic petri dish, lid removed. In the other, the agar was removed from its dish entirely and placed in direct contact with the forest floor. Two other bait types consisted of 1% agar supplemented with 25% Gustavia slurry, made by homogenizing fresh *Gustavia superba* flowers and water in a kitchen blender and then heating the mixture to defaunate it. These agar baits were again either placed in their plastic dishes or first removed from the dish and placed directly on the forest floor. The final two bait types start with the 25% Gustavia slurry recipe. In one case, the agar baits were removed from their dishes and then seeded with fresh, unsterilized Gustavia slurry, to test for the effect of bacterial inoculation from the Gustavia flowers. In the second case, the agar was poured up to the top of the petri dish and then a 1‐mm nylon mesh hot‐glued over the top of the agar. These dishes were then placed mesh‐down on the forest floor, accessible only from underneath through the mesh.

Baits were placed on March 24, 2015, and were collected on March 27, 2015, at which time a sample of the bait was placed on a NGM plate seeded with *E. coli* and the plate was monitored for nematodes twice daily for four days. Twenty‐nine of the 210 baits were absent at the time of collection (in cases we observed, eaten by ants and beetles), leaving data for 181 baits for analysis. From each bait that yielded nematodes, we identified *Caenorhabditis* by morphology and established lines. From each *Caenorhabditis*‐positive sample, we determined the species for at least one line by mating tests.

From 181 baits recovered after three days in the forest, we found 56 (31%) colonized by nematodes, including 17 (9%) colonized by *Caenorhabditis* (15 *C. briggsae* and 3 *C. tropicalis*, including one bait with both species). Colonization rates varied significantly by bait type, for worms overall (*p* < 10^−12^; analysis of deviance from logistic regression), for *Caenorhabditis* generally (*p* = .001), and for *C. briggsae* specifically (*p* < 10^−4^). The worms preferentially colonized baits that made direct contact with the ground over baits that were isolated from the ground by plastic. In both of those classes of bait, the worms preferentially colonized those with peptone enrichments over those with heat‐defaunated *Gustavia superba* flower slurry. And among *Gustavia* plugs, they preferentially colonized those that were not supplemented with raw *Gustavia* slurry. *Caenorhabditis* showed a bait‐type distribution that does not differ significantly from the distribution of baits colonized only by non‐*Caenorhabditis* nematodes (Fisher's exact test, *p* = .92), though the power of this test is limited by the small size of the dataset. Another way to state this is that the probability of *Caenorhabditis* colonizing a bait type is correlated with the probability of only non‐*Caenorhabditis* worms colonizing that same bait type (*r* ^2^ = .98, *p* < .001). This means that *Caenorhabditis* and non‐*Caenorhabditis* nematodes preferred the same baits and colonized each bait type with similar proportions.

### 3.5 Test of colonization by phoresy

We used size‐selective exclosures to determine whether colonization of a resource patch by nematodes requires phoresy on animals of particular sizes. In 2015, we set up arrays of 24 baits in a 6 × 4 grid, with 1 m spacing between samples, at each of six locations spread across BCI. The baits consisted of defaunated *Gustavia superba* flower slurry as described in the previous section. Each array of 24 samples included 4 replicates of 6 different treatments. One treatment consisted of slurry deposited directly onto the forest floor. For the other five treatments, the slurry was placed into a plastic cup, and access to the slurry was restricted by the nature of the cup lid. The lids had a circular opening with 3.1‐cm diameter, which was either totally open or covered with a nylon mesh to restrict access by animals larger than the mesh size. The mesh openings restricted passage to animals smaller than 4, 1, 0.064, or 0.01 mm.

After 5 days in the field, we collected the slurry samples and transferred a small volume (approximately 1 cm^3^) to NGM plates. If worms emerged, we attempted to establish cultures. Surviving cultures were cryopreserved in New York, and species were identified by sequencing and mating tests.

**FIGURE 5:**
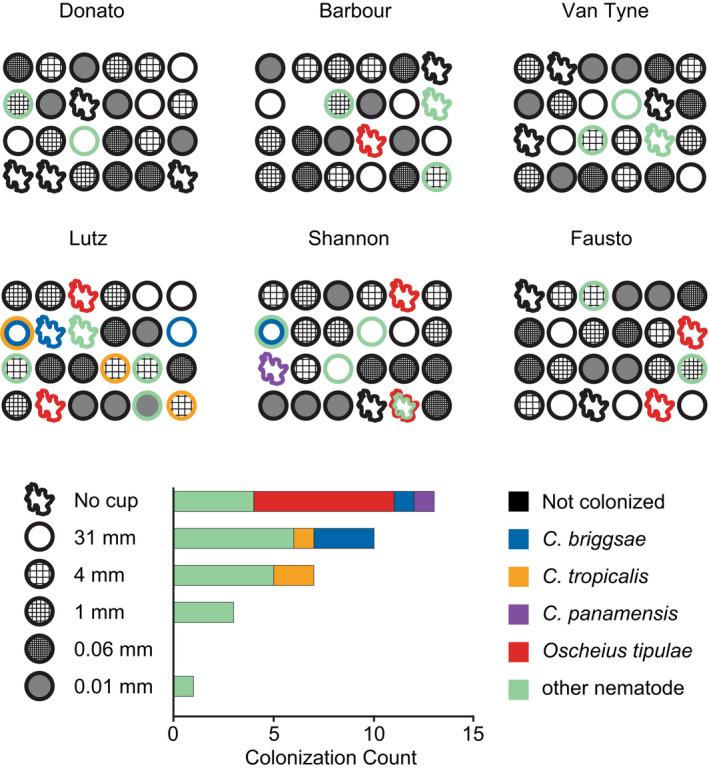
Nematodes colonized 30 baits across six experimental plots, each containing a randomized grid of 4 replicates of each of 6 types of bait differing only in accessibility (143 baits all together with one lost). Accessibility ranged from no barrier to being accessible via 0.01 mm pores. Colonization varied significantly by bait accessibility. *C. tropicalis* and *C. briggsae* both colonized baits isolated from the environment and accessible only by phoresy while *O. tipulae* was only found to colonize baits making direct contact with the ground.

One bait was lost, and of the 143 baits that we recovered, we found nematodes in 30, including three species of *Caenorhabditis* and at least ten additional species (Figure 5; Supporting Information File 1). Because some baits were colonized by multiple species, we count 34 species observations overall.

*C. briggsae* and *C. tropicalis* both colonized baits inside plastic cups, demonstrating that these animals can colonize new substrates by phoresy on other animals. Conversely, *Oscheius tipulae*, which colonized seven baits, only colonized baits that were accessible directly from the soil or leaf litter. Among the animals found in the plastic cups with *Caenorhabditis* were mites, dipterans, hemipterans, coleopterans, and hymenopterans; fly larvae and pupae were common. We observed substantial heterogeneity among the plots (Figure 5). Bait accessibility significantly affected colonization rates by nematodes generally (*p* = 5.4 × 10^−8^; analysis of deviance from logistic regression) and by *Caenorhabditis* specifically (*p* = .007). *Caenorhabditis* colonized only the three most accessible classes of bait, suggesting that their phoretic hosts did not pass through mesh with pores of a millimeter or smaller.

## 4 DISCUSSION

Over the past twenty years, a community effort to study *Caenorhabditis elegans* and its relatives in their natural context has been fruitful. The catalogue of *Caenorhabditis* species and wild isolates has increased dramatically and along with it the ability to apply population, quantitative, and comparative genomic methods (Andersen & Rockman, 2022; Cook et al., 2017; Stevens et al., 2019). Despite these advances, a well‐supported model of any *Caenorhabditis* species' population biology is still missing. Here, we present a deep sampling of *Caenorhabditis* natural diversity in two of the most extensively studied neotropical field sites, along with a collection of experiments aimed at understanding *Caenorhabditis* species ecology and metapopulation structure. In total we collected seven species, four of which were only found in these collections (BCI: *C. becei*, *C. panamensis*, and *C. krikudae* n. sp.; La Selva: *C*. sp. 60). We estimate that we recovered the total number of species at both field sites accessible to our sampling scheme, which was limited by various factors like time of year, selection of visibly rotting material, nematode isolation method, and proximity of sampling localities to trails.

Species from four major clades within *Caenorhabditis* were found in these forests, including representatives of the *Elegans*, *Japonica*, *Angaria*, and *Auriculariae* groups. Our findings comport with biogeographic hypotheses about the history of *Caenorhabditis* diversity (Cutter, 2015). In particular, we find three species (*C. becei*, *C. panamensis*, and *C*. sp. 60) that are part of a neotropical‐endemic clade within the *Japonica* group. Species in this group can be locally abundant in neotropical forests, but their geographic ranges appear to be quite narrow. Each species is known only from a single region, with no overlap among the species in this group found in La Selva, BCI, French Guiana, or Dominica (Marie‐Anne Félix, personal communication; Stevens et al., 2019). Most parts of the neotropics have not yet been surveyed for *Caenorhabditis*, and we infer that many *Japonica* group species remain to be discovered there. Conversely, *Elegans* group species are represented exclusively by two widely distributed androdioecious species, *C. briggsae* and *C. tropicalis*. Endemic gonochoristic *Elegans* group species, which are quite numerous in east Asia and Australia, appear to be absent from the neotropics (personal observations, and Marie‐Anne Félix, personal communication).

Common species at BCI appear to be substrate generalists. Rotten *Gustavia superba* flowers were often occupied by *Caenorhabditis*. We hypothesized that a specific microbial environment on the substrate was preferred by the worms. Our bait preference data suggest that this microbial environment requires conditions that we did not successfully replicate with fresh flower slurry. *Caenorhabditis* species preferred baits supplemented with the general microbial growth medium peptone over the *Gustavia* slurry. Few field studies have looked at substrate preference specifically. Ferrari et al. (2017) found the incidence of *Caenorhabditis* on fresh fruit (citrus) baits to be enriched when compared to non‐*Caenorhabditis* nematodes, while Crombie et al. (2019) concluded that they observed no substrate specificity between *Caenorhabditis* species and their opportunistically sampled substrates. We found that patterns of substrate colonization were highly correlated between *Caenorhabditis* and non‐*Caenorhabditis* nematodes, suggesting that these *Caenorhabditis* communities are substrate generalists. This conclusion is consistent with our opportunistic sampling data, which found no associations between any substrate type and incidence of *Caenorhabditis*.

Our data suggest that *Caenorhabditis* species on BCI disperse by phoresy. In our exclosure experiment, *Caenorhabditis* colonized baits that were directly accessible from the ground, isolated from the ground in a cup, and isolated in a cup and further blocked by mesh with openings of 4 mm or greater. Baits isolated by mesh with openings of 1 mm or smaller were not colonized. These data reinforce the idea of a *propagule pool* model of dispersal in which individuals migrate from a single patch. However, the design of this experiment provides no quantitative measure of the proportion of phoretic versus soil colonizations. In contrast to *Caenorhabditis* species, *Oscheius tipulae* only colonized baits making direct contact with the ground suggesting that they were not colonizing baits using phoresy. While *O. tipulae* is commonly found in soil (Félix, 2006), it was originally isolated from the cadavers of larvae of *Tipula paludosa*, a marsh cranefly (Lam & Webster, 1971). It has also been found more recently on *Rhynchophorus ferrugineus*, a palm weevil (De Luca, 2019). This discrepancy in evidence for phoresy may simply be due to experimental design, bait preference of vector, period of the experiment, or local abundance of vector as a function of geography or seasonality as discussed below.

Species were unevenly distributed over time and geography. There were year‐to‐year changes in the species collected at various localities around BCI. For example, wet season collections at tree DFT yielded *C. briggsae* and *C. becei* in 2012, but during the dry season in 2015, collections at that same tree yielded only *C. tropicalis*. One model is that habitat patches are colonized randomly from the local species pool, as suggested by the patchy species distribution of *G. superba* flower occupancy. An alternative is that species differences among years illustrate ecological succession at larger scales than the level of an individual substrate and its lifespan. Félix and Duveau (2012) more systematically describe seasonal shifts in the abundance of *C. briggsae* and *C. elegans* in a French orchard, paralleling their finding that *C. briggsae* outcompetes *C. elegans* at higher temperatures in the lab. In the neotropics, changes between wet and dry seasons impact the availability of fruit and flower patches, and the availability of phoretic vectors (Leck, 1972).

Species in our spatial sampling dataset appeared to differ in their distributions across sampling sites and quadrats. *C. briggsae* was present in every quadrat at every focal tree sampling site, while other species had a patchier distribution over a scale of meters and at scales between focal tree sampling sites. These patterns could indicate differences in colonization efficiency and differences in the scale of dispersal between species, which might be picked up by a larger dataset. Under the assumption that animals colonize patches independently and randomly, we estimated that about 44% of patches occupied by *C. briggsae* had multiple colonizations. Richaud et al. (2018) modeled *C. elegans* founder number using a Poisson distribution given the proportion of genotypes they observed at a given distance between two patches. They varied how they modeled local haplotype frequencies to account for the unknown proportions of said haplotypes in the source population and came to a mean number of 3–10 founders. Our estimate adds growing support to the hypothesis that colonization event numbers are low for many species across *Caenorhabditis* and that their population biology is affected by living in an ephemeral metapopulation structure. The estimates in our study and in Richaud et al. (2018) are based on androdiecious species. For gonochoristic species at least one individual of each sex must reliably colonize a patch to found a new subpopulation, assuming dispersal is achieved by prereproductive dauer individuals. Founder numbers may be higher for these species while phoresy may ensure that multiple individuals colonize a patch simultaneously. Anecdotally, however, we have on several occasions isolated unmated adult female *Caenorhabditis* from samples that contain no *Caenorhabditis* males, and males from samples that contain no females. Analogously, it has been suggested that the colonization of Réunion island by exclusively hermaphroditic *Pristionchus* species is a likely product of reproductive assurance (Herrmann et al., 2010).

Our data join with comparable field studies in tropical lowland sites in French Guiana and Hawaii to suggest that androdioecious species not only have larger global ranges than dioecious relatives but are also locally dominant (Table 2). Our collection efforts identified *C. briggsae* as the predominant species at BCI followed by *C. tropicalis*, as in lowland Hawaii (Crombie et al., 2019). At La Selva, *C. tropicalis* was the most abundant and the sole dioecious isolate was *C*. sp. 60. This contrasts with the findings at Nouragues, French Guiana, where *C. tropicalis* predominates among the androdioecious species, but the gonochoristic *C. nouraguensis* is the most abundant overall (Ferrari et al., 2017). Taken together, this suggests that the hypothesized benefits of self‐fertile hermaphroditism, including reproductive assurance, population growth advantages, and resistance to Medea elements (Cutter et al., 2019; Noble et al., 2021), are adaptive at multiple spatial scales.

**TABLE 2:** Species counts for samples positive for *Caenorhabditis* collected at four tropical localities. Hawaii Lowlands data are as reported in Crombie et al. (2019), including only samples collected in 2017 from elevations below 500 m. Nouragues data are as reported in Ferrari et al. (2017), representing the count of samples positive for each species summed across collections in 2013, 2014, and 2015.

	BCI	Hawaii lowlands	Nouragues	La Selva
*C. agridulce*	8	0	11	0
*C. astrocarya*	0	0	16	0
*C. becei*	25	0	0	0
*C. brenneri*	0	0	3	0
*C. briggsae*	152	88	37	32
*C. castelli*	0	0	1	0
*C. dolens*	0	0	1	0
*C. kamaaina*	0	2	0	0
*C. krikudae*	2	0	0	0
*C. macrosperma*	0	0	9	0
*C. nouraguensis*	0	0	219	0
*C. oiwi*	0	12	0	0
*C. panamensis*	30	0	0	0
*C*. sp. 60	0	0	0	1
*C. tropicalis*	43	13	178	55

## 5 OUTLOOK

These experiments help inform projects which could more systematically build a model of *Caenorhabditis* species ecology and metapopulation dynamics which includes species co‐occurrence and competition, dispersal dynamics, founding numbers, and the effects of substrate variation and quality. Future studies would best be served by measuring the response of *Caenorhabditis* species incidence to a larger variety of substrate baits and their microbial composition, and the absolute quantity of microbes on those baits to delineate these factors. Sampling potential phoretic vectors using baited exclosure cups along with a deep sampling of soil around common field sites would provide a quantitative model of *Caenorhabditis* colonization strategies. Genetic analysis of field‐collected dioecious individuals at fine spatial and temporal scales would provide data to construct a dispersion kernel to understand dynamics and distance of colonization and estimate founder number while minimizing assumptions about colonization efficiency or source population composition. Understanding modes of dispersal is crucial to understanding patterns of diversity, inbreeding, and selective pressures that metapopulation structure imposes on traits like selfing and sex ratio. Sampling neotropical localities over time will also reveal the spatiotemporal dynamics of the species that coexist there as related to changes in wet and dry seasons, the abundance of patches, and the availability of phoretic hosts.

## AUTHOR CONTRIBUTIONS

**Solomon Sloat:** Conceptualization (equal); data curation (equal); formal analysis (equal); investigation (equal); methodology (equal); project administration (equal); resources (equal); writing – original draft (lead); writing – review and editing (equal). **Luke Noble:** Conceptualization (equal); data curation (equal); formal analysis (equal); investigation (equal); methodology (equal); resources (equal); writing – original draft (equal); writing – review and editing (equal). **Annalise Paaby:** Conceptualization (equal); data curation (equal); formal analysis (equal); funding acquisition (equal); investigation (equal); methodology (equal); resources (equal); writing – review and editing (equal). **Max Bernstein:** Conceptualization (equal); data curation (equal); investigation (equal); methodology (equal); resources (equal); writing – review and editing (equal). **Audrey Chang:** Conceptualization (equal); data curation (equal); investigation (equal); methodology (equal); resources (equal); writing – review and editing (equal). **Taniya Kaur:** Conceptualization (equal); data curation (equal); investigation (equal); methodology (equal); resources (equal); writing – review and editing (equal). **John Yuen:** Conceptualization (equal); data curation (equal); investigation (equal); methodology (equal); resources (equal); writing – review and editing (equal). **Sophia Tintori:** Conceptualization (equal); data curation (equal); investigation (equal); methodology (equal); resources (equal); writing – review and editing (equal). **Jacqueline Jackson:** Conceptualization (equal); data curation (equal); investigation (equal); methodology (equal); resources (equal); writing – review and editing (equal). **Arielle Martel:** Conceptualization (equal); data curation (equal); investigation (equal); methodology (equal); resources (equal); writing – review and editing (equal). **Jose A. Salome Correa:** Conceptualization (equal); data curation (equal); investigation (equal); methodology (equal); resources (equal); writing – review and editing (equal). **Lewis Stevens:** Conceptualization (equal); data curation (equal); formal analysis (equal); investigation (equal); methodology (equal); resources (equal); writing – original draft (equal); writing – review and editing (equal). **Mark Blaxter:** Data curation (equal); funding acquisition (equal); resources (equal); writing – review and editing (equal). **Karin Kiontke:** Data curation (equal); formal analysis (equal); investigation (equal); methodology (equal); resources (equal); writing – original draft (equal); writing – review and editing (equal). **Matthew Rockman:** Conceptualization (equal); data curation (equal); formal analysis (equal); funding acquisition (equal); investigation (equal); methodology (equal); project administration (equal); resources (equal); writing – original draft (equal); writing – review and editing (equal).

## CONFLICT OF INTEREST

None to declare.

## Supporting information


Table S1

Supporting Information File 1

Supporting Information File 2

## Figures and Tables

**FIGURE A1:**
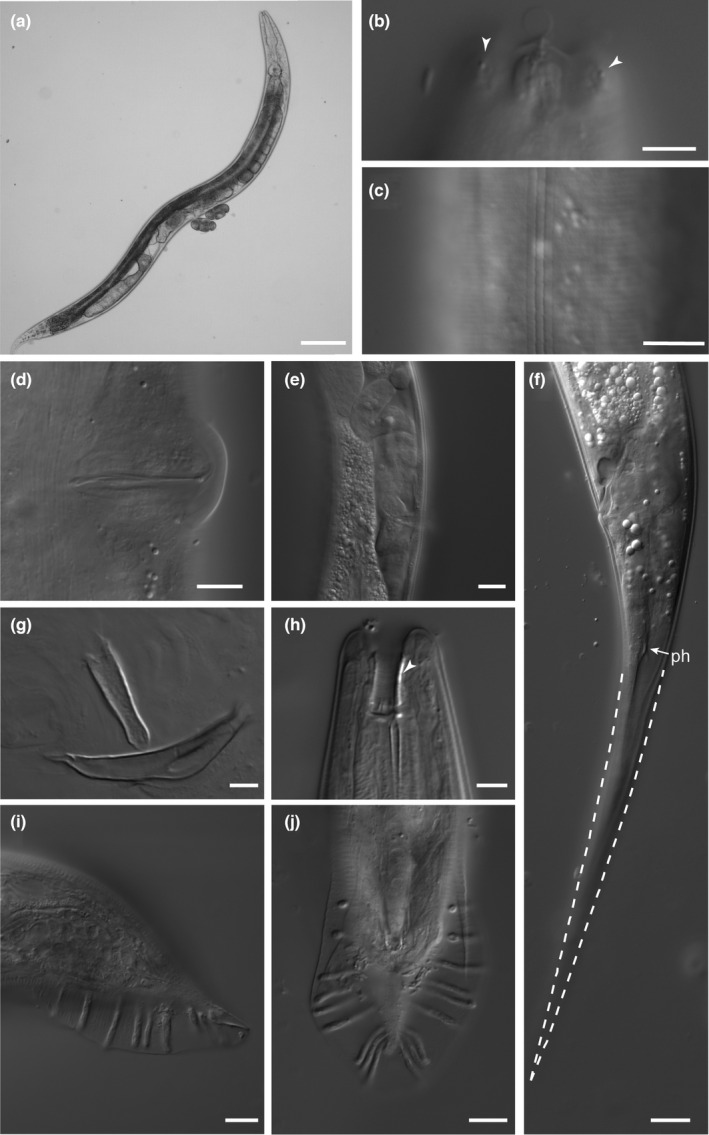
DIC light micrographs of *C. krikudae* n. sp. strain QG3050 female (a,b,d–f) and male (c,g–j) anatomy. (a) Whole adult showing two gonad arms. (b) Left side view of head with cephalic sensilla in focus. (c) alae, (d) vulva, (e) spermatheca, (f) female tail, arrow points to phasmid. (g) Spicule and gubernaculum. (h) Stoma, arrow shows the border between gymnostom and stegostom. Male tail in (i) lateral and (j) dorsal view. Scale bar in (a) is 100 μm; b, g, and h 5 μm; c, d, i, and j 10 μm; e and f 15 μm.

**FIGURE A2:**
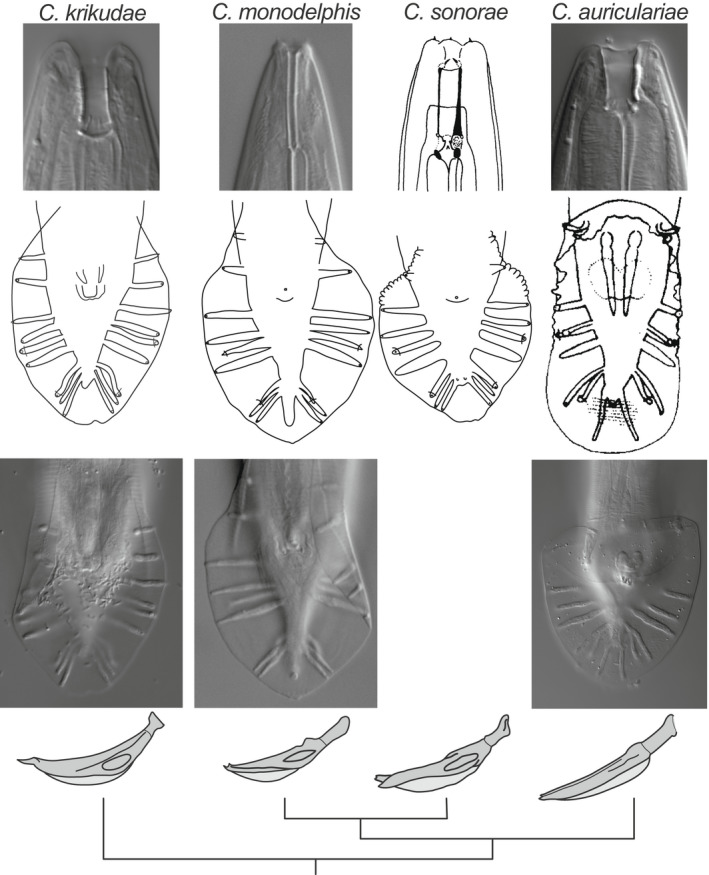
Comparison of stoma, male tail and spicules in species related to *C. krikudae* n. sp. Stoma and male tail morphology differ profoundly in these species. The phylogenetic relationships shown on the bottom are based on this study and Dayi et al. (2021). The position of *C. sonorae* is based on SSU rRNA sequence data only.

**FIGURE A3:**
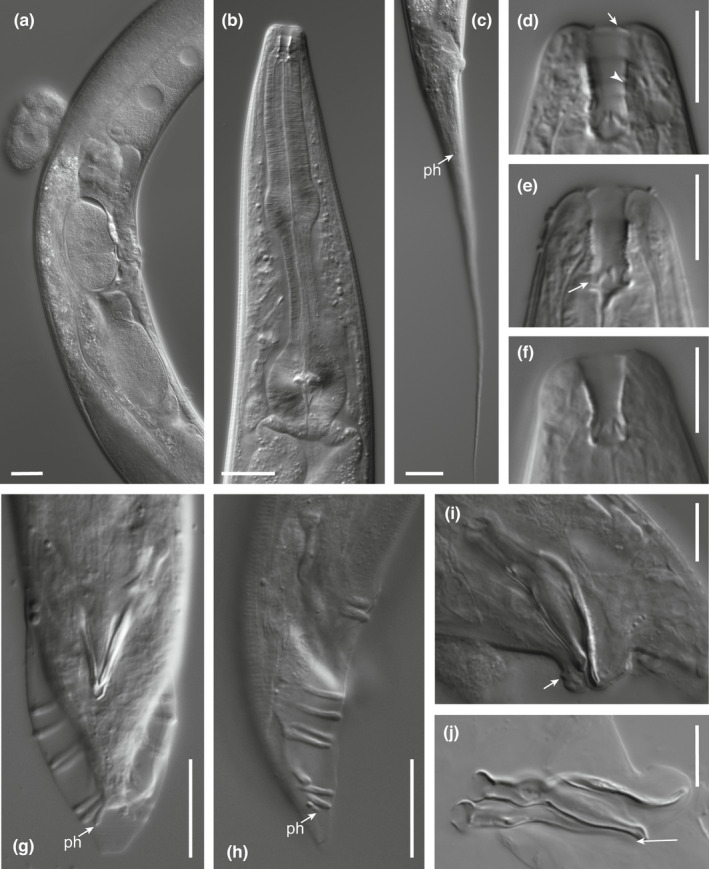
DIC light micrographs of *C. agridulce* n. sp. strain QG555 female (a–e) and male (f–j) anatomy. (a) Vulval region in right side view, showing one embryo in the uterus and one older embryo next to the female. An oocyte is passing through the posterior spermatheca. (b) Pharynx in left side view. (c) tail in right side view. (d) Stoma viewed from a sub‐dorsal right angle; arrow points to cross‐section of the flap on the lip, arrowhead to border between gymnostom and stegostom. (e) Stoma in right side view, arrow points to the dorsal metastegostom; one subventral denticle is also in focus. (f) Male stoma in right side view. (g) Male tail in ventral view; the dorsally directed GP7 on the right side and most GPs on the left side are out of focus. (h) Male tail in right side view. (i) spicules and gubernaculum in situ showing (left side) the precloacal lip (arrow) and its horseshoe‐shaped bulge in cross‐section. (j) Spicules and gubernaculum pushed out of the animal and flattened. Arrow points to the characteristic paw‐shaped spicule tip. ph = phasmid. Scale bars in a, b, c, g, and h 20 μm, in all other images 10 μm.

**FIGURE A4:**
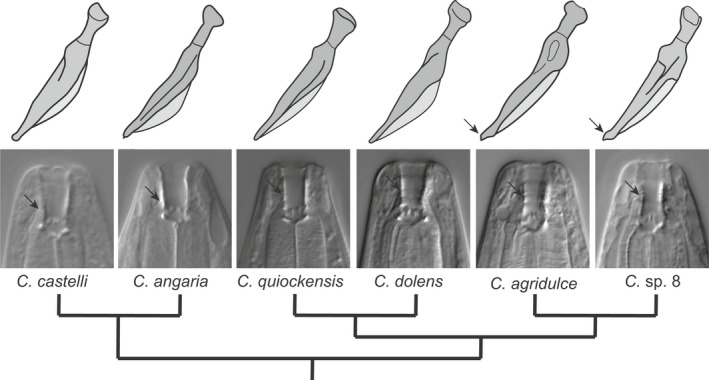
Spicules (drawings) and stomata of species in the *Angaria* group in comparison, and phylogenetic relationships based on this study and an analysis of partial sequences of 15 protein‐coding genes and rRNA genes (Karin Kiontke, unpublished analysis). The spicule tip is more similar in the respective sister species: it is slightly enlarged in *C. angaria* and *C. castelli*, narrow in *C. dolens* and *C. quiockensis*, and paw‐shaped in *C. agridulce* n. sp. and *C*. sp. 8. The stoma is relatively short in all species, but only in *C. angaria* and *C. castelli* is the stegostom unusually short. In all other species, the stegostom and gymnostom contribute at least equally to the stomatal tube; the gymnostom is shorter than the stegostom in *C*. sp. 8. All species display flaps at the lips. The metastegostom carries a projection with a bifid tip (compare Baldwin et al., 1997) in all species. In the light microscope, this structure appears shorter in *C. angaria* and *C. castelli* than in the other species.

## Data Availability

Raw sequencing data and transcriptome and genome assemblies, and annotation files for *C. agridulce* n. sp. have been archived under the ENA study accession PRJEB48807. Raw sequencing data and transcriptome assembly and for *C. krikudae* have been archived under the NCBI study accession PRJNA789856. All collection data are reported in the Supporting Information files.

## APPENDIX A DESCRIPTION OF TWO NEW SPECIES

***Caenorhabditis* nematodes colonize ephemeral resource patches in neotropical forests**

**Methods:** For documentation and comparison of morphological features, we studied isofemales lines QG555 of *C. agridulce* n. sp. and QG3050 of *C. krikudae* n. sp., and *C. auriculariae* Tsuda and Futai (1999) strain NKZ352, *C. castelli* Félix, Braendle and Cutter, 2014 strain JU1426 and *C. dolens* Braendle and Cutter (2017) strain NIC394. The animals were anesthetized in 20 mM sodium azide in diluted M9 buffer and mounted on a pad made with 4.5% Noble Agar in water supplemented with 20 mM sodium azide. The specimens were observed with a Zeiss Axio Imager M2 equipped with differential interference contrast. Photographs were taken at 40x and 100x magnification (Plan Neofluar objectives) with a Zeiss Axiocam 560 Mono and processed either in the Zeiss ZenBlue software or with ImageJ. To better visualize spicule morphology, males were squished between slide and cover glass in a small amount of water and subsequently macerated by drawing 85% lactic acid under the cover glass.

**Species Declarations.**

***Caenorhabditis krikudae* Sloat & Kiontke sp. nov. in Sloat, Noble, Paaby, Bernstein, Chang, Kaur, Yuen, Tintori, Jackson, Martel, Salome Correa, Stevens, Kiontke, Blaxter & Rockman.**

ZooBank identifier urn:lsid:zoobank.org:act:57707388‐B5F0‐4B6D‐B660‐2AFE22BC6811.

= *Caenorhabditis* sp. 57.

**Type material:** 1. Isofemale culture QG3050, cryopreserved as a living stock at the *Caenorhabditis* Genetics Center, Minneapolis, MN. Barro Colorado Island, Panama (coordinates 9.163116667‐79.8372), rotten fig, collected by Matthew Rockman and Solomon Sloat. 2. Isofemale culture QG3050 cryopreserved as a living stock at the NYU Rhabditid Collection. 3. Isofemale culture QG3050, fixed material on slides, deposited at Museo de Invertebrados G. B. Fairchild, Universidad de Panamá.

**Etymology:** This name is derived from kri‐kudé, the word for “tree branch” in the native Panamanian language Guaymí (Alphonse, 1956).

We here describe *C. krikudae* n. sp. based on its distinct DNA barcode sequences, reproductive incompatibility with closely related named species, and distinct morphology. The species reproduces through males and females. This species differs by SSU and ITS2 DNA sequences from all other species in Stevens et al. (2019) and in RhabditinaDB (version beta 0.92, wormtails.bio.nyu.edu, accessed July 3, 2021). In laboratory crosses, QG3050 females do not lay eggs or produce progeny in crosses with *C. monodelphi*s Slos and Sudhaus (2017) strain SB341 males. The females do lay eggs in crosses with *C. auriculariae*, but they are nonviable and die as embryos.

**Type locality ecology and distribution:** The type isolate QG3050 of *C. krikudae* is an isofemale line descended from a mating male and female pair picked to a culture plate on August 26, 2018. These worms were recovered from a Baermann funnel on August 24, after overnight incubation of a rotten fig collected August 23, 2018. The population recovered from the funnel included thousands of nematodes, mostly hermaphroditic *Caenorhabditis*. We subsequently identified two of these worms as *C. briggsae*. A second isolate of *C. krikudae*, QG3065, was recovered from another rotting fig on the same day (Figure 1), where it co‐occurred with non‐*Caenorhabditis* nematodes, one of which matched *Oscheius tipulae* by 18S rDNA sequence.

**Morphology:** (Figure A1).

**Adults:** Cuticle with faint annules under which the struts within the cuticle are visible as dots. Lateral field with three ridges. Lips not offset and not conspicuously fused in pairs. There are no flaps or decorations on the lips. Lip sensilla and amphids are small and inconspicuous. External cephalic sensilla are present in males and females at about the same distance from the lip sensilla as the amphids (Figure A1b arrow). Stoma about as long as the head is wide at the base of the lips. Cheilostom weakly cuticularized. Gymnostom and stegostom each comprising about half of the buccal cylinder. Metastegostom isomorphic and isotopic with a bifid flap on each of the three sides. Pharynx as typical for *Caenorhabditis* with a round median bulb, a double haustrulum in the posterior bulb, and a cardia at the border to the midgut.

**Females:** Vulva a slightly protruding transverse slit, its lateral sides covered with a cuticular flap. A mating plug inconspicuous or absent. The gonads are didelphic and reflexed to the dorsal side as typical. Anterior branch is right, posterior left of the intestine. Both branches do not extend to the level of the vulva. Spermatheca, a S‐shaped tube without conspicuous valves. Copious amount of sperm is visible in the uteri. Sperm diameter ~6 μm, area ~25 μm^2^. The tail is conical and pointed. Phasmid openings are located about 1.5 anal body widths (ABW) posterior of the anus.

**Males:** Testis to the right of intestine ventrally reflexed. Bursa peloderan and anteriorly open. Its edge is smooth, and the terminal end is round. 9 pairs of genital papillae (GP) are arranged as 1 + 1/1 + 3 + 3. The anterior dorsal GP is in position 5 and the posterior dorsal GP in position 7. GP1 and GP3 open at the margin of the velum, thus their tip is not ventrally or dorsally attached. GP6 is not basally enlarged or bottle‐shaped and its tip is not embedded in the cuticle of the velum as in other *Caenorhabditis* species. Instead, it shows a ventrally attached tip in the light microscope. In strain QG3050, GP1 was frequently thin or missing on one side. In one male, GP1 was missing and GP3 was thin. Phasmids are inconspicuous, their opening is on the ventral side of the terminus of the tail. Postcloacal sensilla were not seen with the light microscope. The precloacal sensillum is also very inconspicuous and is rarely seen. The precloacal lip appears as a large smooth flap with a wavy edge towards the cloaca. The gubernaculum is relatively wide and spatula‐shaped with a round distal and a slightly ragged notched proximal end (Figure A1g). The separate, light‐yellow spicules are gently curved with a complex but pointed tip. The dorsal side is weakly cuticularized (“velum”). The proximal third of the blade displays a conspicuous “hole.” The spicule head is small and slightly offset (Figure A2).

**Dauer juveniles:** Long and slender, not ensheathed by the L2 cuticle. Dauer cuticle with prominent annules and broad lateral field. Anus and mouth closed; stoma with conspicuous. metastegostom; pharynx narrow. Amphids and head sensilla not observed, phasmids very inconspicuous. Dauer juveniles wave (nictate) in a spot.

**Comparison with related species:** Based on the molecular phylogeny (Figure 2), *C. krikudae* n. sp. is the sister species of a clade consisting of *C. monodelphis* and *C. auriculariae*. In addition, phylogenetic analyses of rRNA gene sequences place *C. sonorae* (Kiontke, 1997) as the sister species of *C. monodelphis* (Dayi et al., 2021; own unpublished data). Thus, there is likely a clade of four morphologically diverse species. For male tail characters, *C. auriculariae* is particularly distinct. The other three species share several features: an open fan, a large gap between GP1 and GP2, the anterior dorsal GP in position 5 and the posterior dorsal GP in position 7 and a precloacal lip without dramatic decorations. In contrast, *C. auriculariae* has a closed fan, GP1 and 2 are close together; the bases of GP4 and GP5 are very close together in anterior–posterior direction with the base of GP5 being more dorsal than that of GP4, the posterior dorsal GP is in position 8 and the precloacal lip carries a large heart‐shaped appendage (comp. Dayi et al., 2021). Stoma morphology differs dramatically between the four species in this clade. *C. monodelphis* has a long and very narrow stoma without any structures in the area of the metastegostom (the actual presence of which would have to be determined by investigation of the ultrastructure); *C. sonorae* displays a glottoid apparatus with a bulge, similar to that in other rhabditid species, whereas *C. krikudae* n. sp. and *C. auriculariae* have a bifid flap at each side of the metastegostom but no bulge, this feature being common in *Caenorhabditis* species. The lip region in *C. auriculariae* is uniquely different with the lips fused in pairs and each pair carrying a flap that projects into the middle of the mouth, lending its opening a triradiate shape. The position of *C. sonorae* within the *Auriculariae* group implies homoplasy with respect to the 6th genital papilla. *C. krikudae*, *C. auriculariae*, and *C. monodelphis*, GP6 opens with a little tip on the ventral side of the bursal velum, which is the plesiomorphic condition. A GP6 with a tip to the outside is also seen in the fan‐less *C. parvicauda* Stevens and Félix (2019). However, light and SEM observations (Kiontke, 1997) showed that the tip of this GP is embedded in the bursal velum in *C. sonorae*, and this derived trait characterizes all other *Caenorhabditis* species, which form the sister group to *C. parvicauda*.

***Caenorhabditis agridulce* Sloat & Kiontke sp. nov. in Sloat, Noble, Paaby, Bernstein, Chang, Kaur, Yuen, Tintori, Jackson, Martel, Salome Correa, Stevens, Kiontke, Blaxter & Rockman.**

ZooBank ID urn:lsid:zoobank.org: act:461DD591‐E921‐4C0A‐8A93‐E4F9EFB9D4DA

= *C*. sp. 24.

**Type material:** 1. Isofemale culture QG555, cryopreserved as a living stock at the *Caenorhabditis* Genetics Center, Minneapolis, MN. Santa Barbara, California, United States of America (coordinates 34.421629, −119.702021), rotting orange, collected by Annalise Paaby. 2. Isofemale culture QG555 cryopreserved as a living stock at the NYU Rhabditid Collection.

**Etymology:** *C. agridulce* n. sp. is named for the sweet, tart flavor of oranges and *Spondias mombin* fruit.

We describe *C. agridulce* n. sp., based on its distinct DNA barcode sequences, reproductive incompatibility with closely related species, and morphology. The species reproduces through males and females. It is delineated and can be diagnosed by the fertile cross with the type isolate QG555 in both cross directions, yielding highly fertile female and male offspring that are interfertile and cross‐fertile with their parent strains. *C. agridulce* n. sp. differs by SSU and ITS2 DNA sequences from all other species in Stevens et al. (2019) and in RhabditinaDB (version beta 0.92, wormtails.bio.nyu.edu, accessed 3 July 2021). Note that the ribosomal DNA sequences may vary within the species.

From the available sequence data, the named species most closely related to *C. agridulce* n. sp. are *C. quiockensis* Stevens and Félix (2019) and *C. dolens* (Figure 2). In reciprocal laboratory crosses between QG555 and *C. quiockensis* JU2745, females lay embryos but no embryos hatch. On the basis of rDNA sequences, undescribed *C*. sp. 8 (Kiontke et al., 2011) is more closely related to *C. agridulce* n. sp than are *C. quiockensis* and *C. dolens*. Crosses between QG555 males and *C*. sp. 8 QX1182 females produce embryos that arrest and fail to hatch. The reciprocal cross produces dead embryos and a smaller number of small larvae that die as L1.

**Type locality, ecology, and distribution:** The type isolate QG555 is an isofemale line derived from a piece of rotting orange collected from a street tree in a planter on State Street in downtown Santa Barbara on 27 June 2011. The sample also contained *C. elegans* and *Drosophila* larvae.

Other isolates that, based on ITS2 sequences and mating tests, belong to *C. agridulce* n. sp. were isolated from Barro Colorado Island, Panama (this paper), from Nouragues and Kaw Mountain, French Guiana (in Ferrari et al., 2017), and from Los Angeles, California (JU2867), and Tenago, Puebla, Mexico (JU2837) (Marie‐Anne Félix, personal communication). In each case, individuals of the species were found in decaying plant material, mostly fruits.

**Morphology:** (Figure A3).

**Adults:** Medium‐sized species with many characters of the *Angaria* group of *Caenorhabditis*. Cuticle with faint annules and three lateral ridges. Lips not offset or fused in pairs. Ventrally directed flaps on lips (as in *C. angaria* Sudhaus, Kiontke & Giblin‐Davis, 2011) are present. Both sexes with inconspicuous lip sensilla, amphids only rarely visible in the light microscope. Males with conspicuous cephalic sensilla positioned about level with the anterior end of the stomatal tube. Stoma relatively short and occasionally funnel‐shaped. Cheilostom is very weakly cuticularized. Cheilostom and stegostom each contribute about 40% to stoma length in females, gymnostom only about 20%. Stegostom comprises more than half of the stomatal tube, it is thus not as short as in *C. angaria*. Metastegostom with a bifid tooth or flap, which is longer than in *C. angaria*. Pharynx with the round median bulb typical for *Caenorhabditis* and the usual duplex haustrulum and cardia.

**Females:** Vulva a transverse slit with a cuticular flap covering the corners, located in mid‐body. Ovaries amphidelphic with anterior branch to the right and posterior branch to the left of the intestine. Both arms are dorsally reflexed. The reflexed part does not reach beyond the vulva. Spermatheca with prominent sphincters to ovary and uterus. Sperm visible in the uterus, spermatheca, and occasionally in the ovary. The species is oviparous and only few embryos are present in the uteri at a time. The tail is long and conical with a pointed tip. Phasmids located about 2 ABW behind the anus.

**Males:** Testis right of the intestine. The caudal region strongly curved ventrally in dead, anesthetized, and fixed animals, or in males that are submersed. Bursa narrow, peloderan, proximally open, elongated, oval in ventral view with a smooth margin. Nine pairs of genital papillae (GP) and one terminal pair of papilliform phasmids (ph) arranged as (2/1 + 1 + 2 + 3 + ph). Anterior dorsal GP is in position 4, posterior dorsal GP in position 7. GP1 also opens to the dorsal side of the bursal velum; the tip of GP6 is embedded in the cuticle of the bursa, its base thickened. All ventral GPs extend to the edge of the bursa. The phasmids form long papillae with a ventral opening. Precloacal lip with the horseshoe‐shaped bulge as in *C. angaria*. Postcloacal sensilla not observed in the light microscope. Spicules separate, amber‐colored, with a large head, narrow shaft, and straight blade. Dorsal edge of the blade is weakly cuticularized (velum). The tip is widened and shaped like a paw. Gubernaculum narrow, in lateral view with a very slight bulge in the distal third; distal end is curved ventrally, distal and proximal end rounded. Median sperm area is 48 μm^2^ (measured as in Vielle et al. (2016)).

**Comparison with previously described species in the *Angaria* group (**Figure A4 **):** Based on the molecular phylogenies here and in Dayi et al. (2021), *C. agridulce* n. sp. is part of the *Angaria* group of *Caenorhabditis*. *C. agridulce* n. sp. is distinguished from all other species in this group by unique sequences of ribosomal RNA genes. The *Angaria* group also contains sister species *C. angaria* and *C. castelli*, sister species *C. dolens*, *C. quiockensis*, and the undescribed species *C*. sp. 8. A molecular phylogeny with partial sequences of 15 protein‐coding genes and small and large subunit ribosomal RNA (unpublished) places *C. agridulce* n. sp. as the sister species of *C*. sp. 8, and *C. dolens* as the sister species of *C. quiockensis*. Species in the *Angaria* group also share several phenotypic characters that distinguish them from all other *Caenorhabditis* species: The bursa is very narrow, anteriorly open, and oval in ventral view. The mating position is spiral. Males curl their tail around a female, and fixed specimens are conspicuously ventrally curved, suggesting that the diagonal muscles in the tail are adapted to this mating position. The phasmids form papillae. The spicules are amber‐colored and sturdy. Their overall shape is similar in all species, but the shape of the spicule tip varies (Figure A2): It is rounded in *C. angaria* and *C. castelli*, narrower in *C. quiockensis* and *C. dolens* but widened and paw‐shaped in *C. agridulce* n. sp. and *C*. sp. 8. This character distribution is consistent with the hypothesized relationships. In addition, the lips carry flaps that project into the center of the mouth. The stoma is shorter than in most other *Caenorhabditis* species. However, only in *C. angaria* and *C. castelli* is the mesostegostom exceptionally short. In all other species, the stegostom and gymnostom are of about the same length, or the gymnostom is even shorter than the stegostom as in *C. agridulce* n. sp. All species have a bifid tooth on each side of the metastegostom. This structure is small in *C. angaria* and *C. castelli* and slightly larger in all other species.
