#  Corrigendum

**DOI:** 10.1002/ece3.9317

**Published:** 2022-09-14

In the recent article by Sloat et al. (2022), the following sentence was missed in the Acknowledgments section.

“Karin Kiontke was supported by NIH grant GM141395.”

The authors apologize for the error.
