# Supplementary material for: Caenorhabditis nematodes colonize ephemeral resource patches in neotropical forests
Source: Ecol Evol. 2022 Jul 24;12(7):e9124. doi: 10.1002/ece3.9124 (PMC9309040; doi:10.1002/ece3.9124)
Supplement: Supplementary file 3 — Supporting Information File 2 [file ECE3-12-e9124-s002.docx]

Supplementary Table: Phylogenetic Data

TABLE_S1.xlsx contains the list of all species and accessions to genomic/transcriptomic data used in the phylogenetic analysis.

Supplementary File: Collection Data

SupplementaryFile1.xlsx contains all of the collection data reported in the manuscript. Data are provided in a series of sheets corresponding to specific analyses and results, as follows:

**BCI.SamplesBySubstrateAllYears**: Each of the 225 rows records one substrate sample that yielded *Caenorhabditis* nematodes. The species found in each sample are indicated by 1 s in the relevant species columns.

**BCI.Opportunistic2012**: Each row represents an isohermaphrodite or isofemale line established from opportunistic collections in 2012.

**BCI.Spatial2012**: Each row represents an isohermaphrodite or isofemale line established from hierarchical spatial sampling of *Gustavia superba* flowers in quadrats around focal trees in 2012.

**BCI.Exclosures2015**: Each row represents a single bait from one of the 24 baits set out at each of six locations in 2015.

**BCI.Exclusures2015Key**: This sheet provides a key to the columns of the BCI.Exclusures2015 sheet, including descriptions of the exclosure types, coordinates of the exclosures, and a summary of the species represented in each type of exclosure.

**BCI.AgarBaits2015**: Each row represents a single bait from one of the 30 set out at each of seven locations in 2015.

**BCI.AgarBaits2015Key**: This sheet provides a key to the columns of the BCI.AgarBaits2015 sheet, including descriptions of the bait types, and coordinates of the field trials.

**BCI.Opportunistic2018**: Each row records one substrate sample that yielded *Caenorhabditis* nematodes during opportunistic sampling in 2018. The species found in each sample are indicated by 1 s in the relevant species columns.

**LaSelva.Opportunistic2019**: Each row records one *Caenorhabditis* isolate recovered during opportunistic sampling in 2019. Isolates identified by PCR from dried material on Whatman paper have names that start with FTA. Isolates identified from live cultures by mating tests have strain names that start with QG.

**LaSelva.SamplesBySubstrate:** Each row records one substrate sample that yielded *Caenorhabditis* nematodes during opportunistic sampling in 2019. The species found in each sample are indicated by 1 s in the relevant species columns.

**BCI.177Samples2018:** Each row records one substrate sample scored for the presence of nematodes by a single investigator during the 2018 field season. Results of statistical tests for substrate specificity are included.
